# Development of Analytical Method for the Quantitation of Monoclonal Antibodies Solutions via Raman Spectroscopy: The Case of Bevacizumab

**DOI:** 10.3390/ph17040446

**Published:** 2024-03-29

**Authors:** Michail Lykouras, Panagiota Papaspyridakou, Olga E. Makri, Constantine D. Georgakopoulos, Malvina G. Orkoula

**Affiliations:** 1 Institute of Chemical Engineering Sciences, Foundation of Research and Technology-Hellas (ICE-HT/FORTH), GR-26504 Platani, Achaias, Greece; lykouras@iceht.forth.gr; 2 Department of Pharmacy, University of Patras, GR-26504 Rio, Achaias, Greece; 3 Department of Ophthalmology, University of Patras, Medical School, GR-26500 Rio, Achaias, Greece; makriolga@upatras.gr (O.E.M.); cgeorg@upatras.gr (C.D.G.)

**Keywords:** bevacizumab, monoclonal antibody, biopharmaceutical solution, Raman spectroscopy, rotary apparatus, coffee ring, quantitative analysis

## Abstract

Personalized dosages of monoclonal antibodies are being used more regularly to treat various diseases, rendering their quantitation more essential than ever for the right dose administration to the patients. A promising alternative, which overcomes the obstacles of the well-established chromatographic techniques regarding the quantification of biopharmaceuticals, is Raman spectroscopy. This study aimed to develop and validate a novel analytical method for the quantitation of bevacizumab in solutions via Raman spectroscopy. For this purpose, a droplet of the solution was left to dry on a highly reflective carrier and a home-made apparatus was employed for rotation of the sample. Hence, each recorded Raman spectrum was the average of the signal acquired simultaneously from multiple points on a circular circumference. The method was validated, and the detection limit of the antibody was found to be 1.06 mg/mL. Bevacizumab was found to be highly distributed at the formed coffee ring of the dried droplet, though this was a function of solution concentration. Finally, Raman spectra at different distances on the coffee ring were obtained from the four quarters. The lowest bevacizumab detection limit was found at a distance of 75 μm from the external side of the coffee ring and it was determined to be equal to 0.53 mg/mL.

## 1. Introduction

Monoclonal antibodies are protein molecules that are produced by recombinant DNA technology and used as treatment for a wide variety of severe diseases. The use of monoclonal antibodies in the treatment of cancer, chronic inflammatory diseases, autoimmune disorders, asthma, multiple sclerosis, osteoporosis, age-related macular degeneration and infectious diseases has been increasing lately [1,2,3].

A humanized monoclonal antibody used in many cases for treating different types of cancer is bevacizumab, commercially known as Avastin^®^. The action of bevacizumab is based on its binding to the Vascular Endothelial Growth Factor A (VEGF-A). This binding leads to the prevention of the interaction of VEGF-A with its receptor (VEGFR) and the inhibition of the VEGF signalling pathway. Hence, neovascularization and vessel growth are inhibited, and angiogenesis of tumour cells is reduced [4,5]. Thus, bevacizumab is considered an anti-angiogenic monoclonal antibody and is used for the treatment of types of cancer that are characterised by significant angiogenesis. Metastatic colorectal cancer, metastatic breast cancer, non-small-cell lung carcinoma, ovarian cancer, renal cell carcinoma and glioblastoma multiforme are some of the indications for the use of bevacizumab, usually in combination with chemotherapy [6,7].

As far as the dose of bevacizumab is concerned, individualized doses of the monoclonal antibody are prepared according to the prescription for each patient [8]. Moreover, some adverse events of bevacizumab, such as hypertension and proteinuria, are found to be dose-dependent [7,9]. Thus, the quantitation of bevacizumab in the prepared solutions by the healthcare units is of high importance, so that the efficacy and the safety of the anti-angiogenic treatment can be guaranteed. High-performance liquid chromatography (HPLC) is mainly used for the identification and quantification of biological anticancer preparations because of the high specificity and sensitivity and low detection and quantitation limits offered by the method [10,11]. Regarding bevacizumab’s quantitation in Avastin^®^, an HPLC analytical method coupled with mass spectrometry (MS) has been developed and validated [12], while an LC-MS/MS analytical method was recently validated for the quantitation of the monoclonal antibody in human serum [13]. Although the described benefits of the chromatographic techniques are important, they suffer from large time requirements for sample preparations and analysis, requiring specialized personnel, and large amounts of solvents are consumed during the analysis.

Another approach that is gaining ground for the identification and quantification of pharmaceuticals and biomedicals is Raman spectroscopy. Raman spectroscopy is associated with a change in the polarizability of molecules, which is caused by inelastic scattering of the initial monochromatic light with which the substance is irradiated [14,15]. It has been proposed as an alternative to HPLC for the qualitative and quantitative analysis of anticancer drugs and monoclonal antibodies, offering similar accuracy, precision and trueness to the respective analytical validation criteria measured using HPLC [16]. Raman spectroscopy could also serve as a fast, reliable, reproducible, non-invasive, non-destructive, cost-effective and environmentally friendly technique, requiring only a few seconds or minutes for sample preparation, in contrast to HPLC [17]. Thus, the applications of Raman spectroscopy in pharmaceutical and biomedical sciences are impressively increasing [18,19,20,21,22].

A specific application of Raman spectroscopy regarding biopharmaceutical analysis is the identification and quantification of monoclonal antibodies. Only a few studies have been published recently for the characterization and discrimination of monoclonal antibodies using Raman spectroscopy [23,24,25,26,27,28], and the quantification of monoclonal antibodies through Raman spectroscopy has been performed only in very few cases [17,25,29]. In the study of Rayyad et al., three monoclonal antibodies (bevacizumab, trastuzumab and atezolizumab) were quantified in a quartz cuvette and in perfusion bags using Raman spectroscopy [17]. The results of the analytical method developed for the quantification of cetuximab, rituximab, trastuzumab and bevacizumab via Raman spectroscopy using multivariate analysis in another study were found to be significantly affected by the excipients’ concentrations, leading to reliability and reproducibility concerns [25]. Finally, in the study of Le, et al. a machine learning approach for the quantitation of infliximab, bevacizumab, rituximab and ramucirumab in vials has been described and compared to a conventional linear regression approach [29].

The aim of our study was to develop a novel simple, rapid and environmentally friendly analytical method for the quantitative analysis of bevacizumab monoclonal antibody in the final formulation using Raman spectroscopy. The method proposed in this work was also capable of providing automatization for the on-the-spot quantification of bevacizumab in hospitals in the concentration range that is used in the final formulations. The sample preparation method included the drying of a sample droplet on a highly reflective carrier. The acquisition of the Raman spectra was performed by applying rotation on the sample carrier with the dried droplet of the monoclonal antibody solution using a home-made apparatus. A conventional linear regression model was applied for the quantification of the monoclonal antibody in the formulation. It is the first time in the literature that a rotary apparatus has been employed for the quantitation of biopharmaceuticals via Raman spectroscopy, and dehydration of the monoclonal antibody formulation has not been used previously in such an application. A further objective of our study was the validation of the developed quantitative analytical method regarding its specificity, working range, accuracy, precision and sensitivity, as no other study has described a validated Raman spectroscopic method for the quantification of monoclonal antibodies. Finally, our study, also, aimed at the investigation of the optimal position of the dried droplet for Raman spectra acquisition, at which the lowest detection limits could be achieved.

## 2. Results

### 2.1. Identification of Bevacizumab in Avastin^®^ Formulation via Raman Spectroscopy

Avastin^®^ is formulated as a concentrate for solution for infusion, which is packed in 4 mL or 16 mL vials, and the concentration of bevacizumab in the formulation is 25 mg/mL. Apart from the monoclonal antibody, the formulation is composed of trehalose dihydrate, sodium phosphate, polysorbate 20 and water for injections, which are used as excipients. A detailed list of the ingredients, their functions and their concentrations in the formulation are presented in Table 1 [30]. The main excipients of Avastin^®^ are water for injections and trehalose dihydrate, while all other excipients are found in lower concentrations.

**Figure 1:**
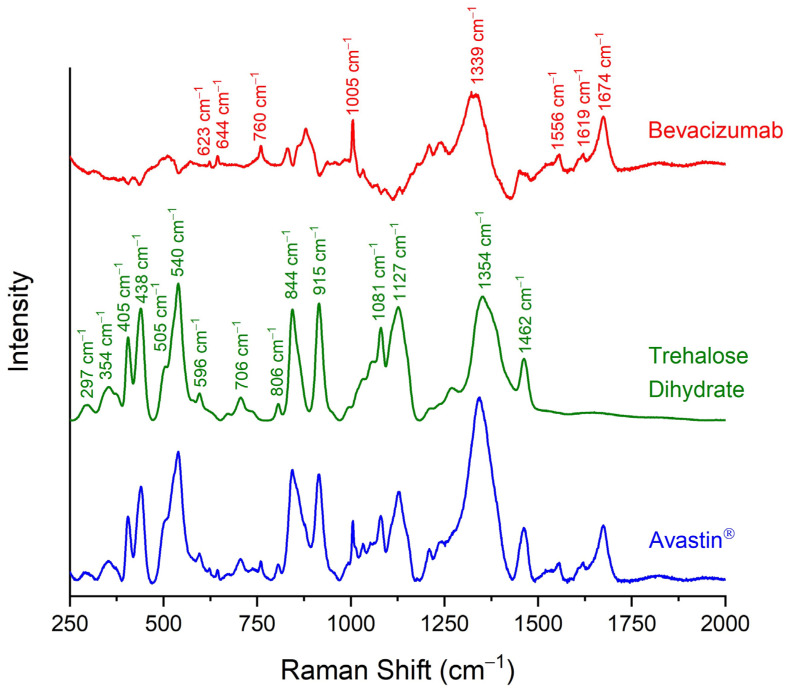
Raman spectra of dehydrated Avastin^®^ formulation (blue line), solid trehalose dihydrate (green line) and pure bevacizumab (red line), as generated by the subtraction of the spectrum of trehalose dihydrate from Avastin^®^. Trehalose (green labels) and bevacizumab characteristic peaks (red labels) in the spectral region of 150–2000 cm^−1^ are indicated.

The Raman spectra of dried Avastin^®^ solution and solid trehalose dihydrate were recorded and they were compared against each other (Figure 1). The peaks of trehalose were easily detected in the Raman spectrum of Avastin^®^. Trehalose dihydrate is characterised by the peaks at 297 cm^−1^, 354 cm^−1^, 405 cm^−1^, 438 cm^−1^, 505 cm^−1^, 540 cm^−1^, 596 cm^−1^, 706 cm^−1^, 806 cm^−1^, 844 cm^−1^, 915 cm^−1^, 1081 cm^−1^, 1127 cm^−1^, 1354 cm^−1^ and 1462 cm^−1^ (Table 2) [17,25,31]. Several extra peaks were observed in the spectrum of Avastin^®^ that did not correspond to trehalose dihydrate. For this reason, the Raman spectrum of trehalose dihydrate was subtracted from the Raman spectrum of the final formulation. The new Raman spectrum (red line in Figure 1) that was generated highly resembled the spectrum of bevacizumab as recorded by Makki, A.A. et al. after isolation of the monoclonal antibody using centrifugal ultrafiltration [25]. Thus, the extra peaks were attributed to bevacizumab, as they are characteristic of monoclonal antibodies and proteins in general. More specifically, the peaks at 623 cm^−1^ and 1005 cm^−1^ were attributed to the phenylalanine (Phe) residues [17,23,24,25,27,28,32], the peak at 644 cm^−1^ to the tyrosine (Tyr) residues [25,26,27,33], the peaks at 760 cm^−1^ and 1556 cm^−1^ were ascribed to the tryptophan (Trp) amino acids [24,25,26,27,34], and the peak at 1619 cm^−1^ was assigned to all three aromatic amino acids [26,32,33,34]. The most prominent peak of bevacizumab, at 1674 cm^−1^, was a depiction of amide I [17,23,24,25,26,27,28], while the peak at 1344 cm^−1^ was attributed to the amide III region, which is usually observed at approximately 1230–1340 cm^−1^ (Table 2) [17,23,25,26,27,28].

### 2.2. Quantification of Bevacizumab in Avastin^®^ Formulation via Raman Spectroscopy

**Figure 2:**
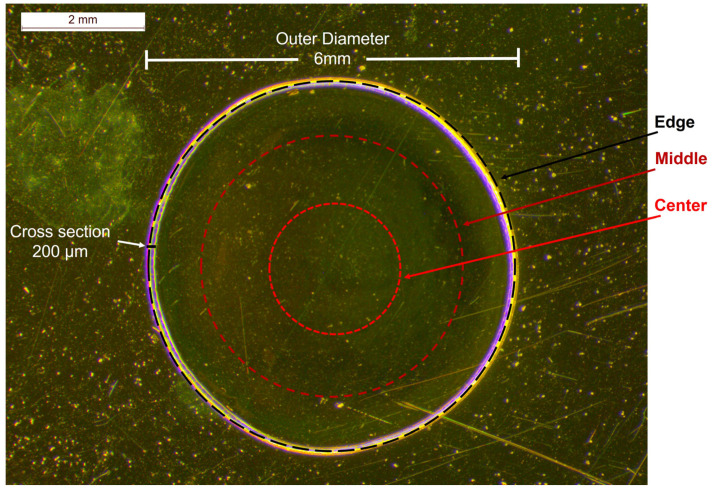
Stereoscopic image of an Avastin^®^ solution drop dried on a highly reflective carrier at RT overnight and the circumferences of circles on which the Raman spectra of the calibration standards were recorded; black dashed circle: edge, dark red dashed circle: middle, red dashed circle: centre.

For the quantification of bevacizumab monoclonal antibody in Avastin^®^ solutions, the initial 25 mg/mL formulation was used as a stock solution, and it was diluted with a sodium chloride (NaCl) 0.9% *w*/*v* solution to prepare calibration standards with concentrations ranging from 3.75 mg/mL to 25.00 mg/mL (3.75 mg/mL, 6.25 mg/mL, 12.50 mg/mL, 18.75 mg/mL and 25.00 mg/mL). A drop of each solution tested (formulation or calibration standards) was placed on a highly reflective gold-coated carrier and dried at room temperature (RT) overnight. A coffee ring, with a radial cross section (thickness) of 200 μm and an outer diameter of approximately 6 mm, was observed under the stereoscope after the dehydration of the droplet (Figure 2).

In order to avoid large errors provided by point irradiation [35,36], a home-made apparatus was employed for rotating the carrier while focusing on the circular circumferences on the dried drop. Hence, the Raman spectra were a result of the average signal of multiple points recorded simultaneously from those circumferences. Three different circles on the dried sample were tested for the acquisition of the Raman spectra of the solution: near the edge of the droplet, close to the coffee ring; at its middle and at its centre (Figure 2).

**Figure 3:**
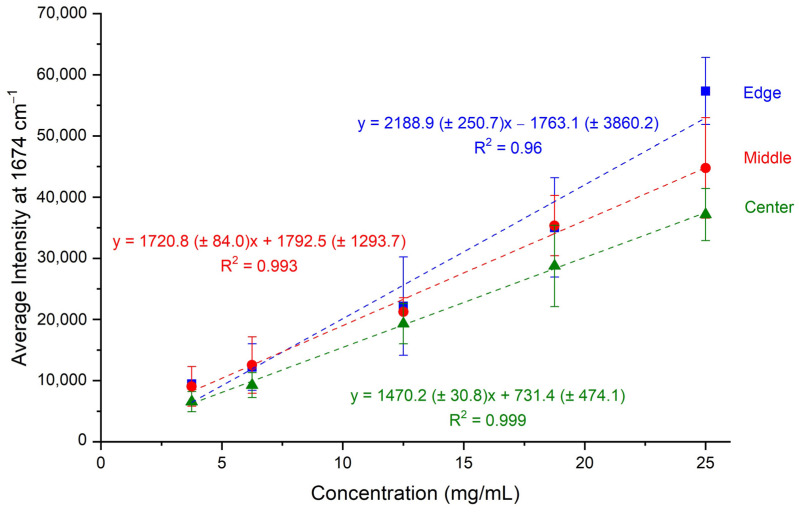
Calibration curves of average intensity of bevacizumab peaks at 1674 cm^−1^ plotted against bevacizumab concentrations for the three different circular circumferences (edge, middle, centre) of Raman spectra acquisition.

The Raman spectra of the calibration standards were recorded in triplicates using the home-made rotary apparatus at the three different circular circumferences. Subsequently, multiple peaks were integrated, and their intensities were determined. The peak at 1674 cm^−1^, the most prominent one attributed to bevacizumab, was finally selected for quantitation purposes. The average intensity of the three sets of dried droplets was calculated for each calibration standard and for each circumference. This average intensity was plotted against the bevacizumab concentration, and three calibration curves were constructed, one for each circle of rotation (Figure 3).

All three calibration curves were rather satisfactory, as their coefficients of determination were close to unity. Especially for the calibration curves created by recording the Raman spectra from the middle and the central circular circumferences, R^2^ values higher than 0.99 were found. The highest R^2^ value was determined for the calibration curve corresponding to the central circle and it was equal to 0.999 (Figure 3). Thus, the three calibration curves were characterised by satisfactory linearity in the working range of 3.75–25.00 mg/mL.

### 2.3. Validation of Developed Quantification Method

The quantification method of bevacizumab using the home-made rotation apparatus was validated regarding its specificity, working range, accuracy, precision, sensitivity and trueness [37].

**Figure 4:**
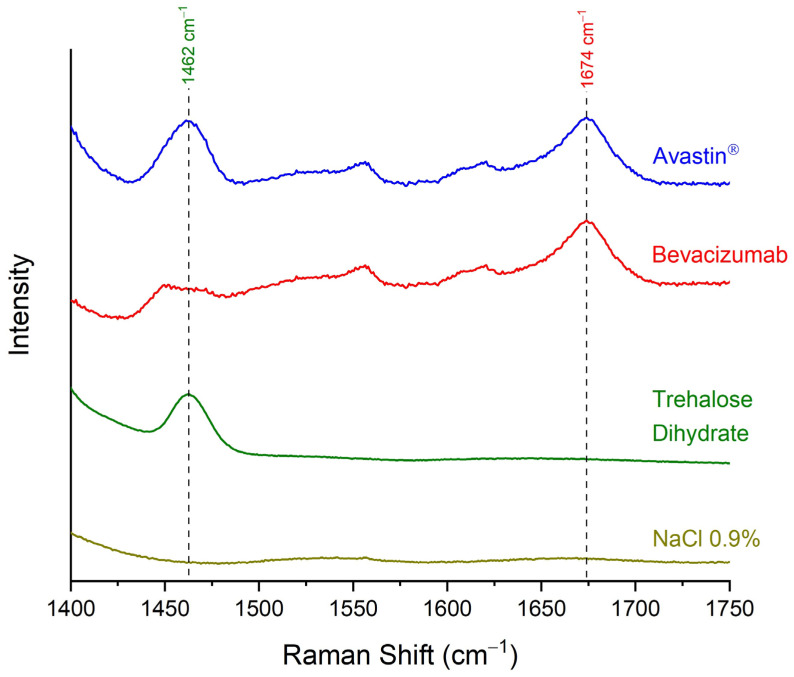
Raman spectra of Avastin^®^ formulation (bevacizumab concentration 25.00 mg/mL), pure bevacizumab, as generated by the subtraction of the spectrum of trehalose dihydrate from Avastin^®^, trehalose dihydrate and blank sample (NaCl 0.9% *w*/*v* solution—bevacizumab concentration 0.00 mg/mL) for validating the selectivity of the method; spectral region: 1400–1750 cm^−1^.

The specificity of the developed quantitative analytical method was tested by comparing the Raman spectra of the initial Avastin^®^ formulation (bevacizumab concentration 25.00 mg/mL), blank sample (NaCl 0.9% *w*/*v* solution—bevacizumab concentration 0.00 mg/mL), trehalose dihydrate and pure bevacizumab, which was the result of subtraction of the Raman spectrum of trehalose dihydrate from the spectrum of Avastin^®^ (Figure 4). The method was found to be specific for the discrimination between bevacizumab and trehalose dihydrate, as no peak of trehalose dihydrate interfered with the peak of the monoclonal antibody at 1674 cm^−1^. Although interference because of the matrix (sample carrier) was observed when comparing Avastin^®^ and NaCl 0.9% *w*/*v* solution Raman spectra, the peak of bevacizumab at 1674 cm^−1^ could be easily discriminated from the noise level (Figure 4). Therefore, the method was found to be selective enough to distinguish among the monoclonal antibody, the excipient and the gold-coated carrier.

Concerning the working range, a satisfactory linearity response in the range of 3.75 mg/mL–25.00 mg/mL was found for all three calibration curves (Figure 3). More specifically, the highest coefficient of determination (R^2^ = 0.999) was achieved for the calibration curve corresponding to the central circular circumference on the dried droplet followed by the calibration curve of the middle circular circumference (R^2^ = 0.993).

The accuracy of the developed method was validated by acquiring the Raman spectra of three sets of dried droplets of a bevacizumab 25.00 mg/mL solution and determining the intensity of the bevacizumab peak at 1674 cm^−1^ of each spectrum for all three positions. The average intensities of the three measurements, as well as the expected intercept values for the 25.00 mg/mL solution according to the calibration curves, were calculated for each circular circumference. The relative errors (E_r_s) of the average intensities compared to the respective expected intensities were less than 15% in all three circular circumferences [38], while for the middle and central ones, the E_r_ was lower than 1% (Table 3); hence, the accuracy of the developed method was satisfactory enough.

Regarding the precision of the developed quantitative method, its repeatability was determined. For this purpose, the intensities of three sets of dried droplets were measured for each circular circumference of sample rotation for three different bevacizumab concentrations: a low concentration (3.75 mg/mL), a middle one (12.50 mg/mL) and a high one (25.00 mg/mL) (Table 4). The average intensities, as well as the respective standard deviations (SDs) and relative standard deviations (RSDs) were calculated for each bevacizumab concentration at each circle. Generally, the maximum accepted RSD values for satisfactory precision are 20% for low concentrations, approaching the QL [38]. However, in the case of low concentrations in biopharmaceutical samples, precision could be considered satisfactory even if only two out of the three RSD values of the different concentrations are inside the accepted values [39]. Thus, the precision of the method was characterised as quite satisfactory, as two RSD out of the three RSD values of the three different concentrations at each rotating position of the sample were below 20% (Table 4).

As far as the sensitivity is concerned, the DL and the QL of the method were determined based on the calibration curves and using the visual evaluation method [37]. The lowest DL (1.06 mg/mL) and QL (3.22 mg/mL) were found for the calibration curve corresponding to the central circular circumference. Low enough also was the DL when Raman spectra were recorded at the middle circular circumference of the droplet (DL = 2.48 mg/mL). In those cases, the results of both methods for the determination of DL were in accordance with each other. However, when the Raman spectra were acquired at the edge of the dried droplet, close to the coffee ring, the DL based on the data of this calibration curve was found to be equal to 5.82 mg/mL, which was significantly higher than the DL estimated by visual evaluation (DL < 3.75 mg/mL) (Table 5). This mismatch at the edge of the dried droplet is caused by the acquisition of the Raman signal not only from points inside the coffee ring but also from points outside the dehydrated drop. Thus, the Raman spectrum at this circumference is an average of the signal from the coffee ring and the gold-coated sample carrier. As a result, a slight underestimation of bevacizumab peak intensity is observed, which is greater for the lowest concentrations. Hence, higher errors are expected in the predicted concentration of bevacizumab at those concentrations.

The trueness of the results predicted by the calibration curves was estimated by using diluted bevacizumab solutions with known concentrations as unknown samples. For this reason, bevacizumab 3.75 mg/mL, 12.50 mg/mL and 25.00 mg/mL solutions were prepared, their Raman spectra were recorded in triplicates by applying rotation at three different circular circumferences after dehydration of a droplet and bevacizumab peak intensities were determined. The average intensities of the three repeats were calculated and bevacizumab concentrations were found from the respective calibration curves. The E_r_s of the calculated bevacizumab concentrations compared to the expected concentrations were determined. The value of the E_r_ should be less than 15% or 20% for low concentrations [38]. Hence, the trueness of the method could be validated for the calibration curves corresponding to the middle and central circular circumference of rotation. For the calibration curve of the circular circumference of rotation at the edge of the dried droplet, the trueness could be validated for the high and medium concentrations, but not for the low one (Table 6).

Therefore, the developed quantification method for recording the Raman spectra of bevacizumab solutions by applying rotation was validated according to its specificity/selectivity, working range, accuracy, precision, sensitivity and trueness, especially for the application of rotation in a middle or central circular circumference of the dried droplet. Some variations from the nominal values of validation were observed when the signal was recorded from the edge circumference of the droplet. These variations should be further investigated because of the fact that the highest bevacizumab intensities were observed at this position.

### 2.4. Investigation of the Optimal Position at the Dried Droplet for Bevacizumab Determination

**Figure 5:**
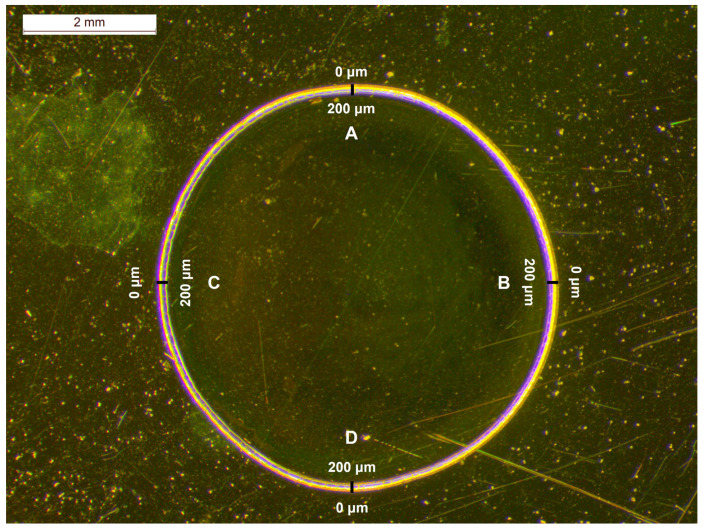
Stereoscopic image of an Avastin^®^ solution drop dried on a highly reflective carrier at RT overnight; the coffee ring, with a radial cross section of 200 μm, was divided into four quarters (A, B, C, D) for recording the Raman spectra.

Bevacizumab could be detected visually in the 3.75 mg/mL calibration standard at the edge circular circumference, although the DL found through the calibration curve was higher (Table 5). Indeed, the intensity of the peak at 1674 cm^−1^ at this position of sample rotation was higher than at the other two circles. As considered, the highest intensities of bevacizumab were detected near the edge of the dried droplet in the coffee ring and, thus, the highest concentration of the monoclonal antibody could be found at this position. When rotation was applied at the edge’s circumference, the laser spot could also record signals from points outside the coffee ring or from internal points of the droplet closer to the middle circumference, because the formed coffee rings were not absolutely circular (Figure 2). Therefore, the quantitation of the monoclonal antibody in the coffee ring was further investigated, as the highest intensities and the lowest DL could be expected in this area of the dried droplet. For this purpose, the dried droplet was divided into four quarters and the optimal position for quantitation was searched for. For each one of them, Raman spectra were obtained, at various distances, initiating at the external edge and finishing at the internal edge of the coffee ring, covering a distance of 200 μm with a step of 25 μm (Figure 5).

**Figure 6:**
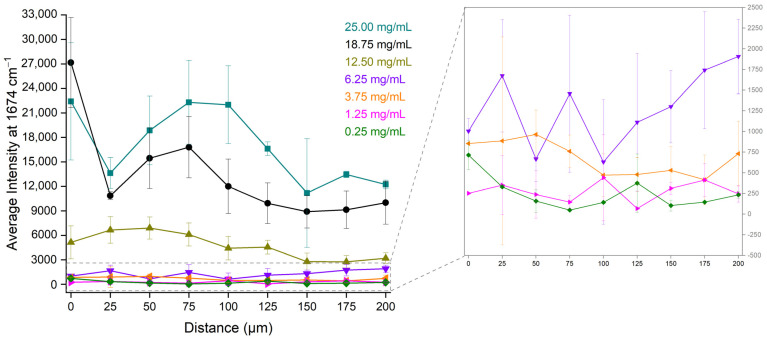
Average Raman intensity of bevacizumab peaks at 1674 cm^−1^ from the four quarters of the dried droplet for the different distances and the different bevacizumab concentrations; insert: magnification of the low bevacizumab concentrations (6.25 mg/mL, 3.75 mg/mL, 1.25 mg/mL and 0.25 mg/mL).

The average intensities of the bevacizumab peak at 1674 cm^−1^ from the four quarters at each distance were calculated. In Figure 6, these average intensities were plotted against the different distances (0 μm–200 μm) from the outer edge of the coffee ring for each bevacizumab concentration. The highest intensity of bevacizumab appeared to follow a specific relation with the distance. For the highest bevacizumab concentrations (25.00 mg/mL and 18.75 mg/mL), the highest intensities were observed near the edge of the drop, for the middle concentrations (12.50 mg/mL and 3.75 mg/mL), they were observed at 50 μm, while for the lowest one (1.25 mg/mL), they were observed at 100 μm. For the 6.25 mg/mL calibration standard, the intensities at 25 μm and 75 μm were also high. Concerning the bevacizumab 0.25 mg/mL solution, no significant differences were found among the intensities of the peaks at 1674 cm^−1^, which were rather low because the monoclonal antibody peaks were either barely detectable or it could not be detected at all. Therefore, the monoclonal antibody was highly distributed closer to the external edge of the coffee ring for its highest concentrations and closer to the middle of the coffee ring, namely at distances between 25 μm and 100 μm, for the middle and the lowest concentrations of bevacizumab (Figure 6).

For the identification of the optimal position for bevacizumab quantitation, the average intensities of the bevacizumab Raman peak at 1674 cm^−1^ from the four quarters of the dried droplet were used. These values were plotted against the respective concentrations of bevacizumab, expressed in mg/mL. For each different distance, a linear fit was applied in a working range of 0.25 mg/mL to 25.00 mg/mL (black dashed lines in Figure S1). Some calibration curves had a satisfactory linear response, such as the calibration curves at 25 μm, 50 μm and 75 μm. However, the coefficient of determination (R^2^) for the rest of the calibration curves was found to be below 0.95, which was probably due to a deviation of the lower concentrations from the linear fit.

Because of the different behaviour of the calibration curves in the low concentrations, the R^2^ values could be improved if this range is studied separately [40]. Thus, one calibration curve was created for the higher (6.25–25.00 mg/mL) (blue dashed lines in Figure S1) and one for the lower bevacizumab concentrations (0.25–6.25 mg/mL) (red dashed lines in Figure S1). The best linear responses for both calibration curves (high and low concentration working ranges) were found for the distances of 25 μm (Figure S1b), 75 μm (Figure S1d) and 125 μm (Figure S1f), while a rather satisfactory linear fit was also determined for both calibration curves at 50 μm (Figure S1c) and 100 μm (Figure S1e). Therefore, for the quantification of bevacizumab, the Raman spectra could be acquired at distances of 25–125 μm from the edge of the dried droplet. For optimal results, the distance of 75 μm from the external side of the coffee ring is suggested (Table 7).

Except for a satisfactory coefficient of determination, the detection limit (DL) and the quantitation limit (QL) at the optimal position should be lower than at the other distances. The DL and QL at each distance were determined based on the calibration curves of the low-concentration working range and through the visual evaluation of the Raman spectra [37]. The lowest DL was found to be equal to 0.01 mg/mL for the distance of 175 μm based on the calibration curve; however, this value could not be verified by visual evaluation of the respective Raman spectra, through which the DL was estimated to be approximately 100 times higher (Table 8). Because of this discrepancy in the DL determined through the two methods, the calibration curve at 175 μm for the low-concentration working range was characterised as insufficient. For the distance of 75 μm, the second-lowest DL was calculated based on the calibration curve equal to 0.53 mg/mL, and it was in quite good compliance with the DL found via the visual evaluation. Moreover, low-enough DLs, approximately 1.5 mg/mL, were also determined for the calibration curves at 25 μm, 50 μm, 100 μm and 125 μm using both methods (Table 8). Thus, the optimal distance from the edge of the dried droplet, providing satisfactory linearity of the calibration curve and a low-enough DL, was found at 75 μm, while these characteristics were also sufficient at a distance range of 25–125 μm from the external side of the coffee ring.

## 3. Discussion

In the present study, a novel analytical method was developed for the quantitation of bevacizumab via Raman spectroscopy. This technique has been recently adopted as a quick, simple and environmentally friendly alternative to the well-established chromatographic techniques, such as high-performance liquid chromatography (HPLC) and electrochemical assays, such as ELISA [41,42,43], for the quantitation of various monoclonal antibodies in their formulations [17,25,29]. This approach involved the initial dehydration of a droplet of the solution on a highly reflective carrier.

The drying of the droplet on a surface is a complicated process, and the final morphology of the dried drop depends on many factors. During this process, the capillary flow causes the transfer of solutes from the main core of the droplet to its contact line, which refers to the edge of the droplet every moment it is drying. This phenomenon triggers the formation of a coffee ring [44,45,46,47]. The dehydration rate is the crucial parameter influencing the shape of the dried droplet, as fast drying leads to homogenous deposition of the solutes, while slower dehydrations possibly result in the formation of a coffee ring deposition. The high surface tension of the solvent, in combination with the surface hydrophobicity, leads to the formation of the coffee ring. Moreover, the formation of the coffee ring is the result of the competition of capillary flow and Marangoni flow [48], which refers to the recirculation of the solutes from the edge to the main core of the droplet. A final parameter that affects the formation of the coffee ring is the viscosity of the solution, promoting the migration of solutes in the droplet. The most viscous solutions hinder capillary flow and promote Marangoni flow, suppressing the coffee-ring effect [44].

The developed method involved the employment of a home-made apparatus for rotating the sample on the gold-coated carrier during Raman spectra acquisition. This method was selected in order to minimize any source of errors in the quantitative analysis due to subsampling when a Raman spectrum from only one point of a solid sample is acquired (point irradiation). It has been widely found through studies that the small size of the laser spot leads to small volumes of sampling and, thus, errors in quantification occur because of the small sampling area and unequal distribution of the sample [36]. Rotation of the sample was proposed as a solution to the errors caused by the unequal deposition of the solute from a dried droplet, as signal from a large number of points (located on the circumference of a circle) is acquired simultaneously. Thus, any issues because of the inhomogeneity of the monoclonal antibody on the dried droplet were reduced.

Three different circular circumferences were tested for obtaining the Raman spectra; the first one was set near the edge of the dried drop, where a coffee ring was formed, the second one in the middle of the dried drop and the third one close to the centre. All three calibration curves possessed a satisfactory linear response in the working range of 3.75–25.00 mg/mL, while the ones corresponding to the middle or central circular circumferences provided R^2^ values higher than 0.990 (Figure 3). When the Raman spectra were acquired under rotation from the centre of the dried droplet, the highest coefficient of determination was found (R^2^ = 0.999), while the standard errors of the slope and intercept had the lowest values (Figure 3) and the DL was the lowest determined (1.06 mg/mL) (Table 4). Low enough also were the DL (2.48 mg/mL) and the standard errors of the calibration curve determined for the middle circle of rotation. These satisfactory linear fits, ensuring low DLs and satisfactory selectivity, accuracy, precision, sensitivity and trueness in the working range of 3.75–25.00 mg/mL, implied an equal distribution of bevacizumab in the main core of the dried droplet, which was independent of the bevacizumab concentration and the exact position of the Raman spectra acquisition.

On the contrary, at the edge’s circular circumference, a lower coefficient of determination (R^2^ = 0.96) was calculated, the standard errors of the slope and intercept were higher than the respective errors of the other two calibration curves and the DL (5.82 mg/mL) (Table S1) was much higher than the DL estimated visually from the respective Raman spectra. Because of the rotation of the sample, it was not possible to focus exclusively on the coffee ring. Hence, from the edge’s circular circumference, the Raman signal was recorded not only from the coffee ring but also from a small area outside the coffee ring and a small area inside the core of the droplet.

Bevacizumab could be detected at all three circular circumferences of the dried droplet. However, the intensity of the monoclonal antibody’s characteristic Raman peak was higher at the edge’s circumference compared to the main core of the dried drop. This behaviour implied that bevacizumab is deposited in the whole area of the dried droplet, but it is highly distributed in the coffee ring. The deposition of the monoclonal antibody in the coffee ring was unequal at different distances and for different concentrations.

Concerning the deposition of the compounds in the coffee ring, their concentrations, their molecular weights and sizes, their solubility and the solvent’s volatility are the most important factors. An unequal distribution of bevacizumab was observed not only among the different areas of the dried droplet, but also in the coffee ring. Hence, the intensity of the most prominent Raman peak of bevacizumab varied significantly according to the distance from the edge of the dried droplet and the concentration of the solute. As a result, the highest intensities of the bevacizumab peak at 1674 cm^−1^ for the 25.00 mg/mL and 18.75 mg/mL bevacizumab solutions were found close to the external edge of the coffee ring, while for the middle and lowest concentrations, they were determined at distances between 25 μm and 100 μm from the outer side of the coffee ring (Figure 6). Thus, bevacizumab was found to be highly distributed between the edge and the middle of the coffee ring (0–100 μm) depending on its concentration.

Although the calibration curves of bevacizumab intensity plotted against the monoclonal antibody concentration offered sufficient coefficients of determination at most of the distances from the edge of the dried droplet (black dashed lines in Figure S1), it was observed that even better linear responses could be achieved if two working ranges were employed [40]; one for the higher (6.25–25.00 mg/mL) (blue dashed lines in Figure S1) and one for the lower bevacizumab concentrations (0.25–6.25 mg/mL) (red dashed lines in Figure S1). In this case, most R^2^ values of both working ranges at all distances had a value over 0.95, implying a satisfactory linearity. Especially at distances between 25 μm and 125 μm, the highest coefficients of determination were observed for both working ranges (Figure S1). For the optimal results, the acquisition of Raman spectra at a distance of 75 μm from the external side of the coffee ring was suggested. At that distance, the highest R^2^ values were determined, and the calibration curves were sensitive enough, as implied by the highest values of slopes, the smallest standard errors of slope and intercept and the low calculated DL (0.53 mg/mL) (Table S2).

According to the summary of product characteristics of Avastin^®^, the initial solution should be diluted with NaCl 0.9% *w*/*v* solution for injection. The final concentration of bevacizumab in the diluted solution should be regulated in a range between 1.4 mg/mL and 16.5 mg/mL according to the patient [7]. The quantification method developed in this study using rotation of the sample offers a linear response in this working range, and the calculated DL for the central circular circumference is lower than 1.4 mg/mL (Table 4). Thus, the bevacizumab concentration in the final solution could be determined successfully by applying rotation on a dried drop. For achieving optimal results and achieving an even lower DL, focusing on the coffee ring at a distance between 25 μm and 125 μm from the external edge is suggested, approximately in the middle of the coffee ring. At 75 μm from the outer side of the coffee ring, the lowest DL was determined, almost 0.5 mg/mL, and the QL was found to be approximately 1.5 mg/mL (Table 6), very close to the lowest concentration used for treatment.

The results of the method developed in our study are in accordance with the results of the studies of Rayyad A. et al. [17] and Makki A.A. et al. [25]. In the first study, bevacizumab was quantified in solution via Raman spectroscopy using a specially designed cuvette with a spherical mirror behind the sample compartment, as well as directly through perfusion bags. The values of the coefficients of determination determined for the middle (R^2^ = 0.993) and the central (R^2^ = 0.999) circular circumferences are close to the respective values found when a quartz cuvette (R^2^ = 0.999) was employed or when bevacizumab was analysed through the perfusion bag wall (R^2^ = 0.999). Moreover, the E_r_ of the predicted concentrations to the expected concentrations and the DLs were similar to the respective values found when the analysis was performed in the quartz cuvette or through the perfusion bag wall [17]. In the latter study, ultrafiltration was applied to the monoclonal antibody solutions, so that bevacizumab would be separated from the excipients and, subsequently, it was identified on the filter. However, bevacizumab was significantly concentrated on the filter, and this concentration is not proportional to the stock solution concentration. Thus, quantitation in this study was performed in the initial monoclonal antibody solutions. The calculated coefficient of determination (R^2^ = 0.995) [25] was comparable to the ones determined in our study and those found in the study of Rayyad A. et al. [17]. In both studies, chemometrics were applied for the quantitative analysis of bevacizumab. These multivariate approaches [17,25], though, did not offer more satisfactory results than the univariate method developed in our study. Moreover, this is the first time in the literature that a combination of the dehydration of a droplet and a rotary apparatus has been applied for the quantitation of a monoclonal antibody.

In another study, various monoclonal antibodies were quantified as solutions in vials via Raman spectroscopy by using a machine learning approach and a conventional linear approach. No significant differences were found between the two methods, although machine learning had an advantage against the conventional linear approach in the prediction of the low concentrations, as the relative errors were significantly reduced. However, the results of machine learning could be biased if the training concentrations are over- or under-sampled [29]. Hence, the linear approaches suggested by the combination of the method employing the home-made rotary apparatus and the method involving focus on the coffee ring developed in our study could be adopted for the quantitative analysis of bevacizumab in the final solutions of Avastin^®^ formulations, as low DLs were predicted and the trueness of the method was satisfactory enough.

## 4. Materials and Methods

### 4.1. Samples

An Avastin^®^ 100 mg/4 mL vial (Roche, Basel, Switzerland) was donated by the Ophthalmology Department of the School of Medicine of Patras University. D-(+)-Trehalose dihydrate, 99% was purchased from Thermo Scientific (Thermo Fisher (Kandel) GmbH, Kandel, Germany) for recording the Raman spectrum of trehalose dihydrate. Sodium chloride (NaCl) 0.9% *w*/*v* 1000 mL solution (Vioser S.A., Taxiarches, Trikala, Greece) was used for the dilutions of the initial Avastin^®^ solution and the preparation of the calibration standards.

The calibration standard solutions were prepared by mixing the appropriate volume of the initial Avastin^®^ 25 mg/mL solution with the respective volume of NaCl 0.9% *w*/*v* solution. Hence, Avastin^®^ solutions with bevacizumab concentrations in a range of 0.25 mg/mL to 25.00 mg/mL were created. As a result, seven standard solutions (0.25 mg/mL, 1.25 mg/mL, 3.75 mg/mL, 6.25 mg/mL, 12.50 mg/mL, 18.75 mg/mL and 25.00 mg/mL) were prepared and used for the construction of the calibration curves. A 10 μL automated pipette (Pipetman Neo^®^, Gilson Inc, Middleton, WI, USA) and a 1 mL automated pipette (BioPette^®^ Autoclavable Pipettes, Labnet International Inc, Edison, NJ, USA) were used for the preparation of the standards. For the homogenization of the standard solutions, shaking in a vortex (MS2 Minishaker, IKA^®^-Werke GmbH & CO., KG, Staufen im Breisgau, Germany) was applied. After the production of the calibration standards, they were analysed immediately and then stored in a refrigerator at 2–8 °C until next use.

### 4.2. Stereoscope

For the observation of the coffee ring of the dried Avastin^®^ droplet, a Stereoscope Leica M80 (Leica Microsystems Ltd., Heerbrugg, Switzerland) with a digital camera (Leica DFC295, Leica Microsystems Ltd., Heerbrugg, Switzerland) was employed. The stereoscope was also equipped with a set of 10×/23B widefield adjustable eyepiece lenses (Leica part number: 10450023, Leica Microsystems Ltd., Heerbrugg, Switzerland) and a 1.0× achromat objective (Leica part number: 10450159, Leica Microsystems Ltd., Heerbrugg, Switzerland). The zoom was set at 1.25×, providing a total magnification of 12.5. The software LAS^©^ V4.13 (Leica Microsystems Ltd., Heerbrugg, Switzerland) was used for capturing the images of the coffee ring of Avastin^®^ droplet dried on the gold-coated carrier.

### 4.3. Raman Spectroscopy

An InVia Raman spectrometer (Renishaw, Wotton-under-Edge, UK) coupled with optical microscope (DM Leica, Leica Microsystems, Wetzlar, Germany) was used for the acquisition of the Raman spectra of Avastin^®^ solutions, trehalose dihydrate and bevacizumab calibration standards. The spectrometer consisted of a 785 nm diode laser for the excitation of the samples, a 1200 gr/mm diffraction grating and a charge-coupled device (CCD) detector. The resolution of the laser was 2 cm^−1^ and its nominal power was 250 mW. For the measurements of the calibration standards, Avastin^®^ solutions and trehalose dihydrate, 90% of the nominal laser power was selected. The time of each Raman spectrum acquisition was set at 10 s, while each spectrum was the result of the accumulation of 10 scans. A 20× (0.4 NA) objective lens (model 566026, Leica Microsystems, Wetzlar, Germany) was employed for the quantitative analysis of the samples. For each Raman spectrum, the spectral area of 100–2000 cm^−1^ was recorded. For the acquisition of the Raman spectra, the Windows-based software WiRE^©^ 2.0 was used.

For the daily calibration of the Raman spectrometer, the Raman spectrum of a silicon (Si) reference standard was recorded before the first measurement of the day. The Raman spectrum of the reference standard was acquired using 1 s as scan time and 5 × 10^−8^% of the nominal laser power, and it was the result of 2 accumulated scans. The calibration of the instrument was validated by the shift of the Si peak at 520 cm^−1^ and the intensity of this characteristic peak.

As sample carrier, a highly reflective gold-coated microscope slide (EMF Corporation, Ithaca, NY, USA) was employed. This sample carrier was a classic microscope slide consisting of two layers; one external bare gold layer (1000 Å) and a second layer of titanium (Ti) (50 Å) binding the external gold layer to the glass of the microscope slide. The slides’ dimensions were 2.6 cm × 7.6 cm and their thickness was 1.0 mm [49]. For the developed quantitative method, each slide was cut into three pieces, so that each slide would be rectangular with a size of approximately 2.6 cm. A droplet (5–10 μL) of each Avastin^®^ solution or calibration standard was placed in the centre of the gold-coated carrier using a 1 mL syringe and left to dry at RT overnight. The rectangular gold-coated carriers were placed on the home-made rotary apparatus and the apparatus was transferred under the microscope of the Raman spectrometer. The point of focus was changed to predetermined distances using the high-precision levers of the microscope stage, and manual focusing correction was applied before the acquisition of each Raman spectrum.

### 4.4. Calibration Curves

After the acquisition of the Raman spectra, the most characteristic peak of bevacizumab at 1674 cm^−1^ was integrated using OriginPro 8^®^ (Originlab Corporation, Northampton, MA, USA). The intensity of the peak was measured after baseline correction in the spectral region of the specific peak [50]. This process was performed for all recorded calibration standards for Raman spectra through both developed quantification methods.

In the developed quantitative method, the average intensity of bevacizumab Raman peaks at 1674 cm^−1^ from three sets of dried droplets was determined at each different circular circumference of rotation. The average intensity of bevacizumab Raman peaks at 1674 cm^−1^ was plotted against the bevacizumab concentration expressed in mg/mL, in order to prepare the calibration curve at each different circumference of the dried droplet (edge, middle, centre). A linear fit was applied to all three calibration curves in a concentration working range of 3.75–25.00 mg/mL.

Concerning the investigation of the optimal position of the dried drop for Raman acquisition, the intensities of the bevacizumab characteristic peak at 1674 cm^−1^ determined from the four sides/quarters at each different distance were averaged. The average intensity of this peak was plotted against the bevacizumab concentration expressed in mg/mL, in order to produce the calibration curve at a distance of 0 μm from the edge of the dried droplet. The same procedure was followed to create the calibration curves at all different distances (0 μm, 25 μm, 50 μm, 75 μm, 100 μm, 125 μm, 150 μm, 175 μm and 200 μm). A linear fit was applied to all calibration curves in a concentration range of 0.25–25.00 mg/mL and two different working ranges; one low-concentration range (0.25–6.25 mg/mL) and another high-concentration range (6.25–25.00 mg/mL).

### 4.5. Validation of the Developed Quantitative Analytical Method

The developed method for quantitating bevacizumab in Avastin^®^ solutions using rotation of the sample carrier was validated for its specificity/selectivity, working range, accuracy, precision, sensitivity and trueness according to the International Council for Harmonization of Technical Requirements for Pharmaceuticals for Human Use (ICH) guidelines for validation of analytical procedures Q2 (R2) [37].

The specificity of the quantification method was validated by the absence of interference of any trehalose peaks or any other peaks due to matrix effects from the sample carrier or the NaCl 0.9% *w*/*v* solution with the bevacizumab peak at 1674 cm^−1^ in the Raman spectra of Avastin^®^ solutions. In case that interference of peaks was observed at 1674 cm^−1^, the selectivity of the method was evaluated by its ability to distinguish between the bevacizumab peak at 1674 cm^−1^ and the interfering peak of the matrix.

Concerning the working range, it was determined by the concentrations of the calibration standard solutions used for the preparation of the calibration curves. A linear relationship between the average intensity of the bevacizumab peak at 1674 cm^−1^ and the bevacizumab concentration was evaluated for the specific working range (3.75–25.00 mg/mL) by the value of the calculated coefficient of determination (R^2^) of the respective regression line. The higher the R^2^ value, the more satisfactory was the linear regression.

The accuracy of this quantitative analytical method was determined by the relative error (E_r_) of the average intensity from three sets of dried droplets of bevacizumab 25.00 mg/mL calibration-standard Raman peaks at 1674 cm^−1^ and the expected values of intensity calculated from the calibration curve for this monoclonal antibody concentration. For a satisfactory accuracy, the E_r_ should not exceed the limit of 15% for the higher concentrations and 20% for the lower concentrations approaching the QL [38].

(1) $$E_{r}\;(\%)=\left|\frac{Average\;Intensity\;at\;1674\;{cm}^{-1}-Expected\;Intensity\;at\;1674\;{cm}^{-1}\;}{Expected\;Intensity\;at\;1674\;{cm}^{-1}}\right|\times 100,$$

Regarding the precision of the developed quantitative method, the repeatability using three sets of dried droplets at three different bevacizumab concentrations was determined. For this purpose, the RSD (%) values of the bevacizumab intensity at 1674 cm^−1^ from three sets of dried droplets were calculated for the bevacizumab 3.75 mg/mL, 12.50 mg/mL and 25.00 mg/mL calibration-standard solutions. The acceptance criteria for satisfactory repeatability involve RSD values lower than 15% for the higher concentrations and lower than 20% for concentrations close to the QL [38]. In the case of biopharmaceutical samples, it is also acceptable even if two out of the three RSD values of the three calibration standards are inside the acceptance criteria [39].

As far as sensitivity is concerned, the detection (DL) and quantitation limit (QL) of the method were determined. Two different approaches were employed for the calculation of the DL and QL. The first method involved the visual evaluation of the DL, i.e., the minimum concentration at which the monoclonal antibody peak at 1674 cm^−1^ could be distinguished visually from the noise level in the Raman spectra. The second approach involved the determination of the DL and QL based on the calibration curve using the following equations:

(2) $$\mathrm{DL}=\frac{3.3\;\times \;\sigma }{m},$$

(3) $$\mathrm{QL}=\frac{10\;\times \;\sigma }{m},$$

where σ is the standard deviation of the response (standard error of y-intercept) and m is the slope of the respective calibration curve [37].

Finally, the trueness of the quantification method was estimated by the prediction of known bevacizumab solution concentrations used as unknown samples in the developed calibration curves. For this purpose, Avastin^®^ solutions with bevacizumab concentrations of 3.75 mg/mL, 12.50 mg/mL and 25.00 mg/mL were prepared, their Raman spectra were recorded from three sets of dried droplets and bevacizumab intensities at 1674 cm^−1^ were measured. Finally, the average intensity of the three measurements was calculated and the concentration of bevacizumab was found from the respective calibration curve. The E_r_ of the determined bevacizumab concentration to the expected bevacizumab concentration was calculated and it should not exceed the value of 15% or 20% for the lowest concentrations [38].

## 5. Conclusions

In the present study, a novel method was developed for the quantification of bevacizumab monoclonal antibody in solutions through Raman spectroscopy. A specific enough method was developed for distinguishing the monoclonal antibody from the excipients in the formulation. Bevacizumab was quantified in dried droplets of the formulation using a home-made rotation apparatus with great success. The application of rotation during Raman spectra acquisition assisted on overcoming any sampling problems due to monoclonal antibody inhomogeneity in the dried droplet. Three different circular circumferences were tested for the application of rotation and the detection limits were calculated for each one. The detection limit could be reduced further if the Raman laser spot was focused on a specific position of the coffee ring, where bevacizumab was more highly distributed. Thus, the deposition of the monoclonal antibody in the coffee ring after the dehydration of the droplet was investigated and the distance range of the highest bevacizumab distribution was determined. The deposition of the monoclonal antibody in the coffee ring was found to be dependent on its concentration. By using this combination of methods, the detection limit was found to be lower than the lowest concentration of bevacizumab in the solutions used for medical treatment. This quantitative analytical method could be also extended for the quantification of other monoclonal antibodies.

## Figures and Tables

**Table 1:** Composition list of Avastin^®^, the function of each ingredient and their concentrations in the vials of the formulations [30].

Ingredient	Function	Concentration (mg/mL)
Bevacizumab	Active Pharmaceutical Ingredient (Monoclonal Antibody)	25.0
Trehalose Dihydrate	Stabilizer	60.0
Monobasic Sodium Phosphate Monohydrate	Buffer	5.8
Dibasic Sodium Phosphate Anhydrous	Buffer	1.2
Polysorbate 20	Surfactant	0.4

**Table 2:** Assignment of the Avastin^®^ trehalose dihydrate and bevacizumab Raman spectrum peaks [17,23,24,25,26,27,28,32,33,34].

Raman Shift (cm^−1^)	Trehalose Dihydrate	Bevacizumab
297	O-C-C bending vibration	-
354	O-C-C bending vibration	-
405	deformation in the plane of glucopyranose ring	-
438	deformation in the plane of glucopyranose ring	-
505	CH_2_ rocking deformation and O-C-C bending mode	-
540	O-C-C bending vibration	-
596	C-C-O bending vibration	-
623	-	phenyl ring breathing modes of Phe
644	-	phenyl ring breathing vibrations of Tyr
706	deformation in the plane of glucopyranose ring	-
760	-	benzene and pyrrole rings breathing modes of Trp
806	CH_2_ twisting vibration	-
844	C-O-C skeletal mode and C-C stretching vibration	-
915	C-O stretching	-
1005	-	phenyl ring angular bending vibrations of Phe
1081	C-O stretching	-
1127	C-O, C-C stretching and deformation in the plane of glucopyranose ring	-
1339	-	amide III NH bending and Cα-N stretching vibrations
1354	CH_2_ rocking deformation	-
1462	CH_2_ scissoring mode	-
1556	-	C=C stretching vibration of the phenyl ring of Trp
1619	-	phenyl ring bond-stretching vibrations of Phe, Tyr and Trp
1674	-	amide I C=O stretching of carbonyl groups

**Table 3:** Accuracy of the quantification method for the three different circular circumferences.

Bevacizumab 25.00 mg/mL	Edge	Middle	Centre
1st Set Intensities	61,761.8	35,341.6	34,518.3
2nd Set Intensities	59,013.1	50,575.5	34,889.5
3rd Set Intensities	51,232.6	48,317.0	42,069.5
Average Intensities	57,335.9	44,744.7	37,159.1
Expected Intensities	52,959.4	44,812.5	37,486.4
E_r_ (%)	8.26	0.15	0.87

**Table 4:** Precision—repeatability of the quantification method for the three different circular circumferences.

Circular Circumference of Rotation	Bevacizumab Concentration (mg/mL)	Intensities					RSD (%)
		1st Set	2nd Set	3rd Set	Average	SD	
Edge	3.75	9409.4	9981.7	8976.1	9455.7	504.4	5.33
	12.50	31,311.5	19,181.3	16,038.6	22,177.1	8065.1	36.37
	25.00	61,761.8	59,013.1	51,232.6	57,335.9	5461.3	9.53
Middle	3.75	11,973.9	9632.9	5582.0	9062.9	3233.8	35.68
	12.50	23,891.7	20,102.3	19,776.1	21,256.7	2287.8	10.76
	25.00	35,341.6	50,575.5	48,317.0	44,744.7	8221.2	18.37
Centre	3.75	6113.6	8384.0	5186.1	6561.2	1645.3	25.08
	12.50	22,914.2	18,608.8	16,439.3	19,320.8	3295.6	17.06
	25.00	34,518.3	34,889.5	42,069.5	37,159.1	4256.6	11.46

**Table 5:** Detection limits (DLs) and quantitation limits (QLs) of bevacizumab for the different circular circumferences based on the calibration curves and through visual evaluation.

Circular Circumference of Rotation	Calibration Curve Method		Visual Evaluation Method
	DL (mg/mL)	QL (mg/mL)	DL (mg/mL)
Edge	5.82	17.64	<3.75
Middle	2.48	7.52	<3.75
Centre	1.06	3.22	<3.75

**Table 6:** Trueness of the quantification method for the three different circular circumferences.

Circular Circumference of Rotation	Expected Bevacizumab Concentration (mg/mL)	Calculated Bevacizumab Concentration (mg/mL)	E_r_ (%)
Edge	3.75	5.125	36.67
	12.50	10.937	12.50
	25.00	26.999	8.00
Middle	3.75	4.225	12.66
	12.50	11.311	9.51
	25.00	24.960	0.16
Centre	3.75	3.965	5.74
	12.50	12.644	1.15
	25.00	24.777	0.89

**Table 7:** Calibration curves, coefficient of determination (R^2^) values, DLs and QLs for the different distances on the coffee ring.

Distance (μm)	Working Range (mg/mL)	Calibration Curve	R^2^
0	0.25–6.25	y = 150.4 (±52.1)x + 140.4 (±222.4)	0.89
	6.25–25.00	y = 1175.7 (±177.6)x − 7625.3 (±2937.4)	0.98
25	0.25–6.25	y = 264.5 (±29.2)x − 21.3 (±124.6)	0.99
	6.25–25.00	y = 641.1 (±56.3)x − 1814.5 (±963.8)	0.98
50	0.25–6.25	y = 241.9 (±47.0)x + 30.3 (±107.5)	0.96
	6.25–25.00	y = 1009.9 (±110.3)x − 5314.7 (±1887.9)	0.98
75	0.25–6.25	y = 262.4 (±9.8)x − 196.0 (±41.8)	0.999
	6.25–25.00	y = 1171.1 (±128.8)x − 6635.2 (±2204.2)	0.98
100	0.25–6.25	y = 82.3 (±8.5)x + 134.5 (±35.9)	0.99
	6.25–25.00	y = 1146.9 (±157.8)x − 8155.5 (±2700.6)	0.96
125	0.25–6.25	y = 208.7 (±25.4)x − 229.9 (±108.5)	0.99
	6.25–25.00	y = 829.8 (±82.3)x − 4917.0 (±1408.4)	0.98
150	0.25–6.25	y = 187.8 (±35.1)x + 21.5 (±129.9)	0.93
	6.25–25.00	y = 572.5 (±98.1)x − 2900.9 (±1679.0)	0.94
175	0.25–6.25	y = 266.0 (±0.3)x + 79.9 (±1.1)	1.0
	6.25–25.00	y = 664.0 (±118.3)x − 3603.5 (±2024.8)	0.94
200	0.25–6.25	y = 332.2 (±79.6)x − 284.2 (±339.7)	0.95
	6.25–25.00	y = 603.9 (±116.2)x − 2601.3 (±1988.9)	0.93

**Table 8:** Detection limits (DLs) and quantitation limits (QLs) of bevacizumab at different distances from the outer edge of the coffee ring based on the calibration curve and visual evaluation methods.

Distance (μm)	Calibration Curve Method		Visual Evaluation Method
	DL (mg/mL)	QL (mg/mL)	DL (mg/mL)
0	4.88	14.79	3.75
25	1.55	4.71	1.25
50	1.47	4.45	1.25
75	0.53	1.59	0.25
100	1.44	4.36	1.25
125	1.71	5.20	1.25
150	2.28	6.92	1.25
175	0.01	0.04	1.25
200	3.37	10.23	1.25

## Data Availability

Data is contained within the article.

## Supplementary Materials

The following supporting information can be downloaded at: https://www.mdpi.com/article/10.3390/ph17040446/s1, Figure S1: Calibration curves at the whole calibration standards concentration range (0.25–25.00 mg/mL, black dashed lines), at the high (6.25–25.00 mg/mL, blue dashed lines) and at the low concentration working range (0.25–6.25 mg/mL, red dashed lines) of the average intensity of bevacizumab peaks at 1674 cm^−1^ plotted against bevacizumab concentration at distances of (**a**) 0 μm; (**b**) 25 μm; (**c**) 50 μm; (**d**) 75 μm; (**e**) 100 μm; (**f**) 125 μm; (**g**) 150 μm; (**h**) 175 μm; (**i**) 200 μm from the edge of each dried droplet; Table S1: Calibration curves, coefficient of determination (R^2^) values, DLs and QLs for the three different circular circumferences (summarized results of the rotation method); Table S2: Calibration curves, coefficient of determination (R^2^) values, DLs and QLs for the different distances on the coffee ring (summarized results of the quarters method).
