# Supplementary material for: Development of Analytical Method for the Quantitation of Monoclonal Antibodies Solutions via Raman Spectroscopy: The Case of Bevacizumab
Source: Pharmaceuticals (Basel). 2024 Mar 29;17(4):446. doi: 10.3390/ph17040446 (PMC11055105; doi:10.3390/ph17040446)
Supplement: Supplementary file 1 [file pharmaceuticals-17-00446-s001.zip › pharmaceuticals-2876751-supplementary.pdf]

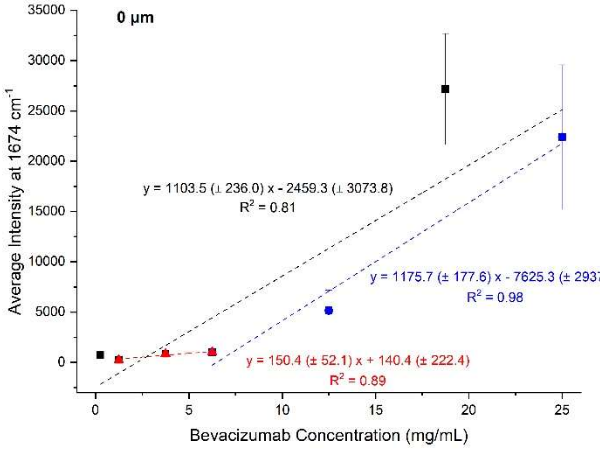
0 µm
y = 1103.5 (± 236.0) x - 2459.3 (± 3073.8)
R² = 0.81
y = 1175.7 (± 177.6) x - 7625.3 (± 2937.4)
R² = 0.98
y = 150.4 (± 52.1) x + 140.4 (± 222.4)
R² = 0.89
Average Intensity at 1674 cm⁻¹
Bevacizumab Concentration (mg/mL)

(**a**)

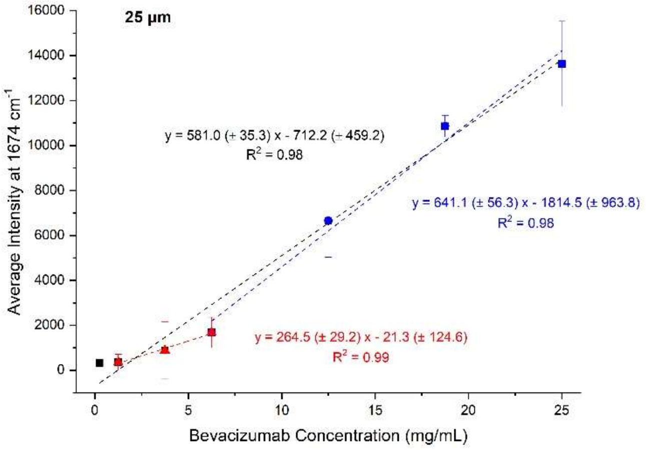
25 µm
y = 581.0 (± 35.3) x - 712.2 (± 459.2)
R² = 0.98
y = 641.1 (± 56.3) x - 1814.5 (± 963.8)
R² = 0.98
y = 264.5 (± 29.2) x - 21.3 (± 124.6)
R² = 0.99
Average Intensity at 1674 cm⁻¹
Bevacizumab Concentration (mg/mL)

(**b**)

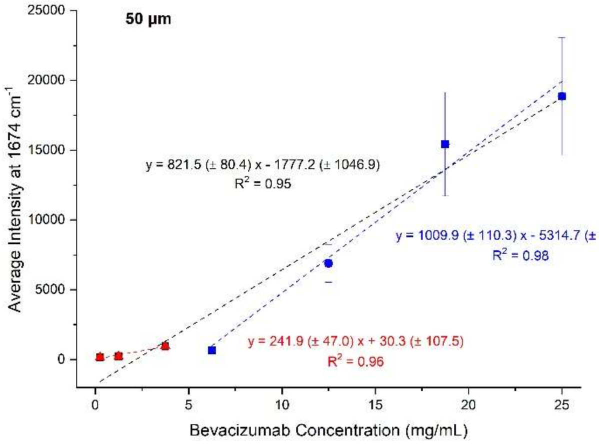
50 µm
y = 821.5 (± 80.4) x - 1777.2 (± 1046.9)
R² = 0.95
y = 1009.9 (± 110.3) x - 5314.7 (± 1887.9)
R² = 0.98
y = 241.9 (± 47.0) x + 30.3 (± 107.5)
R² = 0.96
Average Intensity at 1674 cm⁻¹
Bevacizumab Concentration (mg/mL)

(**c**)

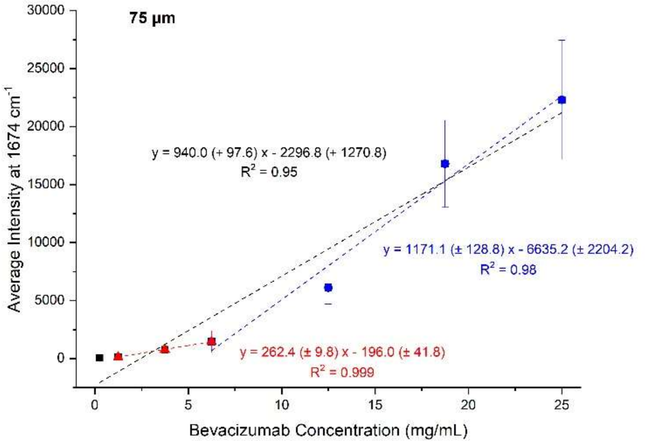
75 µm
y = 940.0 (± 97.6) x - 2296.8 (± 1270.8)
R² = 0.95
y = 1171.1 (± 128.8) x - 6635.2 (± 2204.2)
R² = 0.98
y = 262.4 (± 9.8) x - 196.0 (± 41.8)
R² = 0.999
Average Intensity at 1674 cm⁻¹
Bevacizumab Concentration (mg/mL)

(**d**)

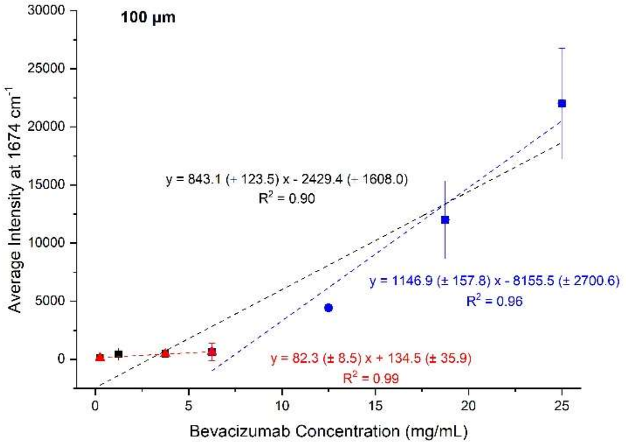
100 µm
y = 843.1 (± 123.5) x - 2429.4 (± 1608.0)
R² = 0.90
y = 1146.9 (± 157.8) x - 8155.5 (± 2700.6)
R² = 0.96
y = 82.3 (± 8.5) x + 134.5 (± 35.9)
R² = 0.99
Average Intensity at 1674 cm⁻¹
Bevacizumab Concentration (mg/mL)

(**e**)

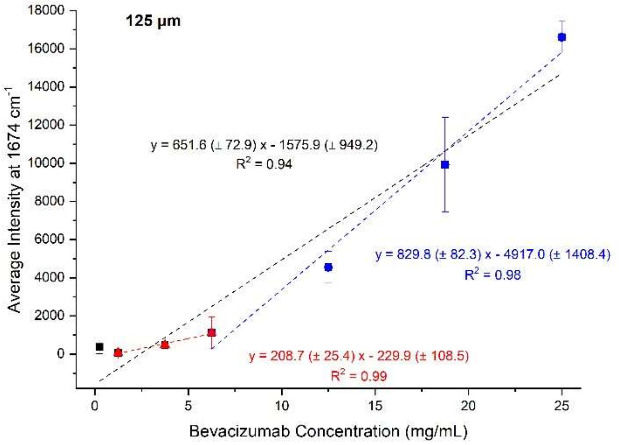
125 µm
y = 651.6 (± 72.9) x - 1575.9 (± 949.2)
R² = 0.94
y = 829.8 (± 82.3) x - 4917.0 (± 1408.4)
R² = 0.98
y = 208.7 (± 25.4) x - 229.9 (± 108.5)
R² = 0.99
Average Intensity at 1674 cm⁻¹
Bevacizumab Concentration (mg/mL)

(**f**)

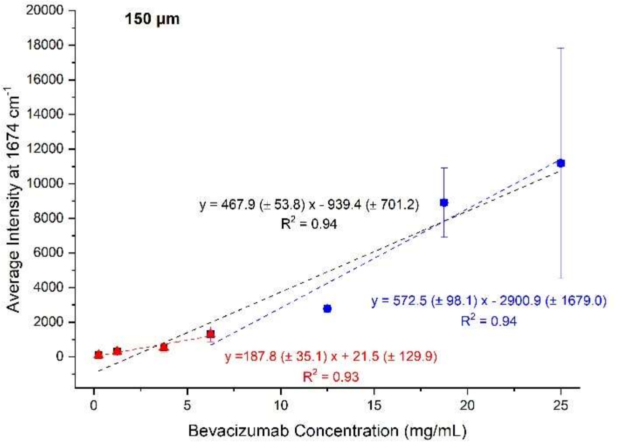

(**g**)

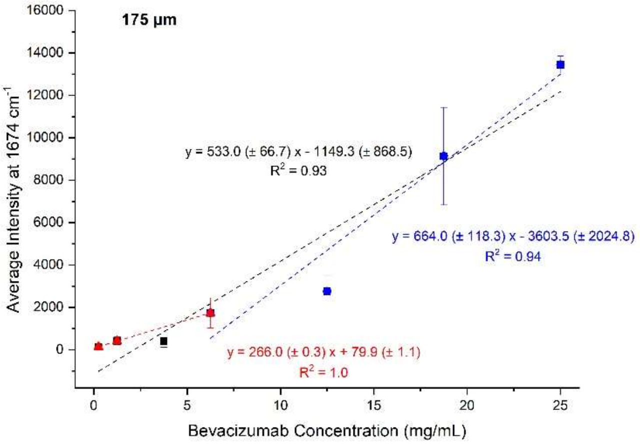

(**h**)

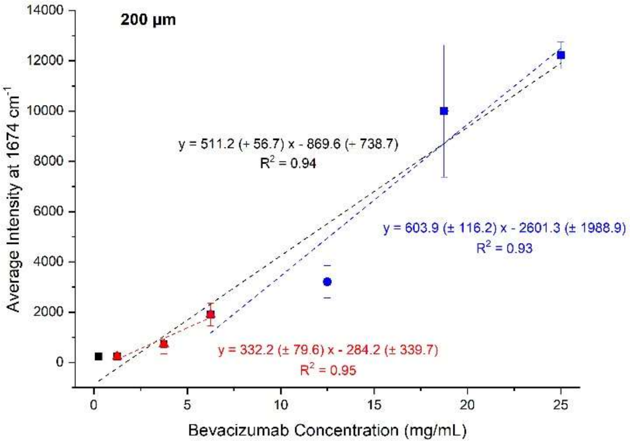

(**i**)

Figure S1. Calibration curves at the whole calibration standards concentration range (0.25 mg/mL – 25.00 mg/mL, black dashed lines), at the high (6.25 mg/mL – 25.00 mg/mL, blue dashed lines) and at the low concentration working range (0.25 mg/mL – 6.25 mg/mL, red dashed lines) of the average intensity of bevacizumab peaks at 1674 cm^{-1} plotted against bevacizumab concentration at distances of (**a**) 0 µm; (**b**) 25 µm; (**c**) 50 µm; (**d**) 75 µm; (**e**) 100 µm; (**f**) 125 µm; (**g**) 150 µm; (**h**) 175 µm; (**i**) 200 µm from the edge of each dried droplet.

Table S1. Calibration curves, coefficient of determination (R^2) values, DLs and QLs for the three different circular circumferences (summarized results of the rotation method)

Circular Circumference of Rotation	Calibration Curve	R^2	DL (mg/mL)	QL (mg/mL)
**Edge**	y = 2188.9 (± 250.7) x – 1763.1 (± 3860.2)	0.96	5.82	17.64
**Middle**	y = 1720.8 (± 84.0) x + 1792.5 (± 1293.7)	0.993	2.48	7.52
**Center**	y = 1470.2 (± 30.8) x + 731.4 (± 474.1)	0.999	1.06	3.22

Table S2. Calibration curves, coefficient of determination (R^2) values, DLs and QLs for the different distances on the coffee ring (summarized results of the quarters method)

Distance (μm)	Working Range (mg/mL)	Calibration Curve	R^2	DL (mg/mL)	QL (mg/mL)
**0**	0.25 – 6.25	y = 150.4 (± 52.1) x + 140.4 (± 222.4)	0.89	4.88	14.79
	6.25 – 25.00	y = 1175.7 (± 177.6) x – 7625.3 (± 2937.4)	0.98		
**25**	0.25 – 6.25	y = 264.5 (± 29.2) x – 21.3 (± 124.6)	0.99	1.55	4.71
	6.25 – 25.00	y = 641.1 (± 56.3) x – 1814.5 (± 963.8)	0.98		
**50**	0.25 – 6.25	y = 241.9 (± 47.0) x + 30.3 (± 107.5)	0.96	1.47	4.45
	6.25 – 25.00	y = 1009.9 (± 110.3) x – 5314.7 (± 1887.9)	0.98		
**75**	0.25 – 6.25	y = 262.4 (± 9.8) x – 196.0 (± 41.8)	0.999	0.53	1.59
	6.25 – 25.00	y = 1171.1 (± 128.8) x – 6635.2 (± 2204.2)	0.98		
**100**	0.25 – 6.25	y = 82.3 (± 8.5) x + 134.5 (± 35.9)	0.99	1.44	4.36
	6.25 – 25.00	y = 1146.9 (± 157.8) x – 8155.5 (± 2700.6)	0.96		
**125**	0.25 – 6.25	y = 208.7 (± 25.4) x – 229.9 (± 108.5)	0.99	1.71	5.20
	6.25 – 25.00	y = 829.8 (± 82.3) x – 4917.0 (± 1408.4)	0.98		
**150**	0.25 – 6.25	y = 187.8 (± 35.1) x + 21.5 (± 129.9)	0.93	2.28	6.92
	6.25 – 25.00	y = 572.5 (± 98.1) x – 2900.9 (± 1679.0)	0.94		
**175**	0.25 – 6.25	y = 266.0 (± 0.3) x + 79.9 (± 1.1)	1.0	0.01	0.04
	6.25 – 25.00	y = 664.0 (± 118.3) x – 3603.5 (± 2024.8)	0.94		
**200**	0.25 – 6.25	y = 332.2 (± 79.6) x – 284.2 (± 339.7)	0.95	3.37	10.23
	6.25 – 25.00	y = 603.9 (± 116.2) x – 2601.3 (± 1988.9)	0.93		
